# Acoustic cloak based on Bézier scatterers

**DOI:** 10.1038/s41598-018-30888-7

**Published:** 2018-08-27

**Authors:** Zhimiao Lu, Lorenzo Sanchis, Jihong Wen, Li Cai, Yafeng Bi, José Sánchez-Dehesa

**Affiliations:** 1 Wave Phenomena Group, Department of Electronic Engineering, Universitat Politècnica de València, Camino de vera s.n., ES-46022 Valencia, Spain; 2 Vibration and Acoustics Research Group, Science and Technology on Integrated Logistics Support Laboratory, National University of Defense Technology, Changsha, 410073 P.R. China; 3 Key Laboratory of Noise and Vibration Research, Institute of Acoustics, Chinese Academy of Sciences, Beijing, 100190 China

## Abstract

Among the different approaches proposed to design acoustic cloaks, the one consisting on the use of an optimum distribution of discrete scatters surrounding the concealing object has been successfully tested. The feasibility of acoustic cloaks mainly depends on the number and shape of the scatterers surrounding the object to be cloaked. This work presents a method allowing the reduction of the number of discrete scatterers by optimizing their external shape, which is here defined by a combination of cubic Bézier curves. Based on scattering cancellation, a two-dimensional directional cloak consisting of just 20 Bézier scatters has been designed, fabricated and experimentally characterized. The method of fundamental solutions has been implemented to calculate the interaction of an incident plane wave with scatterers of arbitrary shape. The acoustic cloak here proposed shows a performance, in terms of averaged visibility, similar to that consisting of 120 scatterers with equal circular cross sections. The operational frequency of the proposed cloak is 5940 Hz with a bandwidth of about 110 Hz.

## Introduction

Acoustic cloaking is a topic of increasing interest since the application of transformation techniques into acoustics, which has allowed the design of cloaking shells rendering objects undetectable to sound waves^1–4^. The designed shells ideally reroute the incident wave to avoid the interaction with the object. One type of shells consists of metamaterials with spatial-varying anisotropic density mass anisotropy and bulk modulus. Artificial structures with such unusual properties have been proposed^5^ but their practical realization becomes extremely difficult. However, simplification approaches have been employed to design reduced cloaks that have been validated experimentally. For example, Zhang *et al*.^6^ created a cylindrical cloak shell for underwater application by using a network of acoustic circuit elements. Carpet cloaks for two-dimensional (2D) and three-dimensional (3D) objects have been also demonstrated using periodic arrangements of perforated plastic plates^7,8^. Similar materials were employed to create the illusion of an empty cavity^9^. A different type of shells obtained from transformation acoustics involve the use of metamaterials with scalar density but with a highly anisotropic bulk modulus named *pentamode* materials^10^. Recently, Chen *et al*.^11^ demonstrated a pentamode cloak consisting of 500 hexagonal unit cells for underwater operation.

The main advantage of transformation cloaks reported so far are their relative broadband operation. However, the complexity associated with the practical realization of the artificial structures with the required properties, make them unfeasible. Due to the difficulties in building the anisotropic materials resulting from transformation methods, a cloak design method called scattering cancellation were proposed for electromagnetic waves^12^ and extended to acoustic waves^13–16^.

The scattering cancellation approach make use of a coating that cancels the scattered field from the composite object, thereby eliminating the scattering from the original object. Using classic scattering theory, the coatings can be design by direct methods^12,17–19^, or by inverse approaches, using optimization algorithms^14–16^. Cloaking by optimization was proposed for electromagnetic waves^20^ and further experimentally confirmed^21^. Based on optimization, acoustic cloaks made of a finite number of individual scatters were designed, providing the position and size of the scatterers. Topology optimization applied to scattering cancellation^22^ has the advantage of providing the material distribution within the coating but its practical realization uses to be difficult. Experimentally, demonstrations have been performed with 2D and 3D obstacles in air for a narrow band of frequencies and one-directional operation, using coatings based on simple scatterers like cylinders with circular cross-section^14^ and toroids^15^, respectively. For the case of objects in a water background, recently, the scattering cancellation of hollow cylinders were demonstrated by using an an elastic coating^19^. Another interesting devices, like flat acoustic lenses^23^ have been also designed using optimization algorithms. First, a successful demonstration was performed using a collection of scatterers with simple sections^24,25^. Later on, this approach was extended using scatterers in which their external shape was also optimized^26^.

Directionality and narrow band operation are drawbacks associated with the concealing coatings based on discrete scatterers^14,15^ In order to overcome these drawbacks, the design procedure based on collection of scatterers with simple shapes requires a large number of units. Therefore, the reduction of the number of scatterers defining an axisymmetric cloak is a must if one wants to obtain omnidirectional coatings with broadband operation.

This work reports the design, construction, and experimental demonstration of an axisymmetric acoustic cloak in 2D with a minimum number of scatterers. The reduction of the number of scatterers is achieved by incorporating the optimization of the scatterer shape into the design procedure. A combination of Bézier curves is employed to build the external shape of the scatterers. Therefore, the size, position and shape of the scatterers defining the coating surrounding a rigid cylinder are obtained by developing an optimization procedure, where the parameters defining the Bézier curves are embedded in the scattering cancellation condition. The optimum design consists of 20 Bézier scatterers, providing a performance in terms of the visibility factor similar to that reported in ref.^14^ using 120 scatterers.

## Results

### Design procedure

In designing the coating that conceals the scattering of a cylindrical obstacle with circular section, we consider that the obstacle or object to be cloaked is acoustically rigid as the majority of solids are in air. For practical reasons, the cloak will be designed for an object with circular section of radius *R*_0_ = 5.625 cm and for a working frequency of 6000 Hz; i.e., at a wavelength *λ* ≈ *R*_0_. However, the procedure here explained is general and can be applied to cylindrical obstacles with any arbitrary section and for any desired frequency. The main drawback associated to the single-frequency design procedure followed in this work is the narrow band operation of the resulting cloak. In order to increase the bandwidth operation, the optimization procedure could be improved by considering additional frequencies simultaneously. In this case, the procedure should be reformulated by defining a multi-objective fitness function. However, the design of broadband cloaks presents its own technical issues that are out of the scope of the present work.

The cloak consists of a certain number of cylindrical scatterers, also acoustically rigid, but with non-circular sections. In fact, the cross-section of each scatterer is obtained as a result of the optimization process. The section is obtained using a type of topology optimization in which the shape is determined by the flexibility provided by the combination of two cubic Bézier curves^27^.

It should be pointed out that losses are not an issue for these cloaks based on scatterers with rounded shapes, like circular and Bézier curves. Numerical simulations recently performed in the framework of Boundary Element Method (BEM) and Finite Element Method (FEM) predict that visco-thermal effects decrease the scattered signal in small amounts; from 5% to 7%^28^.

**Figure 1:**
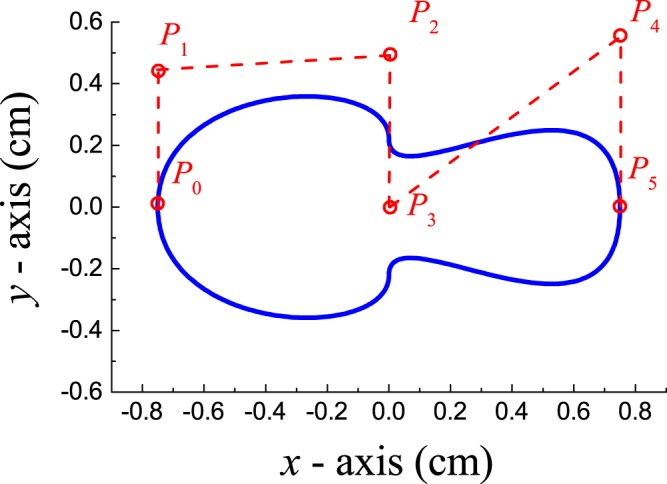
Representation of the cross-section of a typical Bézier cylindrical scatterer forming the cloak here designed. The blue line defines its external profile, which is obtained from the six control points **P**_*i*_ (*i* = 0 to 5). The coordinates of these points depend on five parameters that are obtained from the optimization process involving the scattering cancellation condition.

The scatterer shape is defined by six control points, **P**_*i*_, as it is shown in Fig. 1, where the blue continuous line defines its external profile. Two cubic Bézier curves are employed in it construction. The first one, **B**_1_, is built with the first four control points **P**_*i*_ (*i* = 0, 1, 2, 3) while the second one, **B**_2_, is built with the control points **P**_*i*_ (*i* = 2, 3, 4, 5). Thus, the parametric definition of the profile contained in the semi-plane $y\ge 0$ (see Fig. 1) is given by

1 $${{\bf{B}}}_{1}(\varsigma )={\mathrm{(1}-\varsigma )}^{3}{{\bf{P}}}_{0}+3\varsigma {\mathrm{(1}-\varsigma )}^{2}{{\bf{P}}}_{1}+3{\varsigma }^{2}\mathrm{(1}-\varsigma ){{\bf{P}}}_{2}+{\varsigma }^{3}\frac{{{\bf{P}}}_{2}+{{\bf{P}}}_{3}}{2},$$

2 $${{\bf{B}}}_{2}(\varsigma )={(1-\varsigma )}^{3}\frac{{{\bf{P}}}_{2}+{{\bf{P}}}_{3}}{2}+3\varsigma {(1-\varsigma )}^{2}{{\bf{P}}}_{3}+3{\varsigma }^{2}(1-\varsigma ){{\bf{P}}}_{4}+{\varsigma }^{3}{{\bf{P}}}_{5},$$

where *ς* varies within the interval [0, 1]. In addition, the profile contained in the semi-plane $y\le 0$ is obtained from the expressions above by applying the mirror operation *y* → − *y*.

Compared with scatterers with circular section, the Bézier scatterers provide additional degrees of freedom (DOF) to control the acoustic scattering and, therefore, gives the possibility of achieving the acoustic cancellation condition with a lower number of scatterers.

The coordinates of the six control points defining a single Bèzier scatterer depend on five parameters that are obtained as a result of the optimization process. As a typical example, Table 1 gives the relationship between four of the five parameters and the coordinates of the control points determining a single Bézier scatterer, the one represented in Fig. 1. Three of them, *a*_*i*_ (*i* = 1, 2, 3), determine the shape of the scatterer. The parameter *β* determines its length, 2 *βr*_0_, with *r*_0_ being an arbitrary fixed value. The fifth parameter, not shown in Table 1, corresponds to the angle of tilting *θ* of the scatterer with respect to the *x*−axis. For the scatterer shown in Fig. 1, *θ* = 0°.

**Table 1:** Cartesian coordinates of the six control points, **P**_*i*_, employed in the construction of the Bézier scatterer shown in Fig. 1. They are given in terms of the parameters employed in the optimization process: *a*_1_, *a*_2_ and *a*_3_ are the shape parameters, *β* is a scale factor and *r*_0_ is an arbitrarily fixed value. The blue curve in Fig. 1 is obtained with the following data: *a*_1_ = 0.278, *a*_2_ = 0.295, *a*_3_ = 0.36, *β* = 1 and *r*_0_ = 0.75 cm.

coordinates	P_0_	P_1_	P_2_	P_3_	P_4_	P_5_
x	−*βr*_0_	−*βr*_0_	0	0	*βr*_0_	*βr*_0_
y	0	2*a*_1_*βr*_0_	2*a*_2_*βr*_0_	0	2*a*_3_*βr*_0_	0

In total, a given scatterer within the cloak is defined by these five parameters together with its two spatial coordinates, all of them are obtained at the end of the optimization process.

### Optimization method

An optimization method that combines the genetic algorithm (GA)^29^ and the simulated annealing (SA)^30^ have been developed to obtain the shape and size of the scatterers. The combination of both optimization algorithms overcomes the respective limitations of GA and SA when used independently. The combined algorithm is capable of reaching a solution closer to the global minimum. For more details of the optimization method the reader is addressed to ref.^31^, where the method was introduced and applied in designing optical cavities with ultrahigh Purcell factor. In the case under study here, instead of changing the position of the cylindrical scatterers, the GA-SA method moves the coordinates of the six **P**_*i*_ points on the plane.

The optimization procedure starts by fixing the number of Béziers scatterers employed to get the concealing of the central obstacle, a cylindrical object with circular cross section in this case. The positions and the shape at each position of the corresponding scatterer are obtained as a result of the optimization process.

As the fitness function, *F*, in the optimization process we employ the following expression (in polar coordinates),

3 $$F={[1+\frac{\sum _{i=1}^{360}{|{p}^{sc}({r}_{c},{\theta }_{i})|}_{cloak}}{\sum _{i=1}^{360}{|{p}^{sc}({r}_{c},{\theta }_{i})|}_{object}}]}^{-1},$$

where (*r*_*c*_, *θ*_*i*_) are the polar coordinates of 360 points uniformly distributed in a circumference with radius *r*_*c*_ in the far field; i.e., at *r*_*c*_ = 0.75 m. The expression involves the calculation of total scattered pressure amplitudes, $|{p}^{sc}|$ resulting from the interaction of an incident plane wave traveling along the positive *x*-axis with the bare *object* (located at the origin of coordinates) and with the object surrounded with the *cloak*. *F* can take values $F\le 1$. The value F = 1 defines the complete acoustic concealment, where the perfect wave front is recovered and the numerator is equal to zero. After optimization, the configuration with the highest *F* provides the positions and shape of the scatterers defining the optimum cloak.

The directional symmetry of the cloak implies a strong reduction of the optimization problem. Thus, we need to determine the parameters of only one fourth of the cylinders in the cloak. The rest are determined by the symmetry operations associated to the two mirrors planes involved in the structure. For example, for a cloak containing *N* scatterers Bézier, only the parameters of the *N*/4 scatterers contained in one quadrant need to be determined. Since a given scatterer is defined with seven parameters, in principle, the design algorithm involve the determination of *N*/4 × 7 parameters, *N*/2 of them corresponds to the coordinates of the *N*/4 scatterers. However, in order to reduce the computing effort, we consider that all the scatterers have the same shape; i.e., *a*_1_, *a*_2_ and *a*_3_ are equal. Therefore, the number of parameters to be optimized is reduced to *N* + 3. Thus, for a cloak containing 20 scatterers, the number of parameters to be optimized is reduced to 23. To verify that we have found a global rather than a local minimum, we run the optimization algorithm with different initial conditions.

### Optimum cloak

The optimization procedure described above has been employed to design cloaks with a different number of Bézier scatterers. For the sake of comparison, the optimization algorithm has been also applied to design cloaks based on scatterers with circular cross-section. Two different approaches have been followed for this case; one in which the circular section of all the scatterers have the same radius and another in which the algorithm has the flexibility of optimizing the scatterer radius at each position.

**Figure 2:**
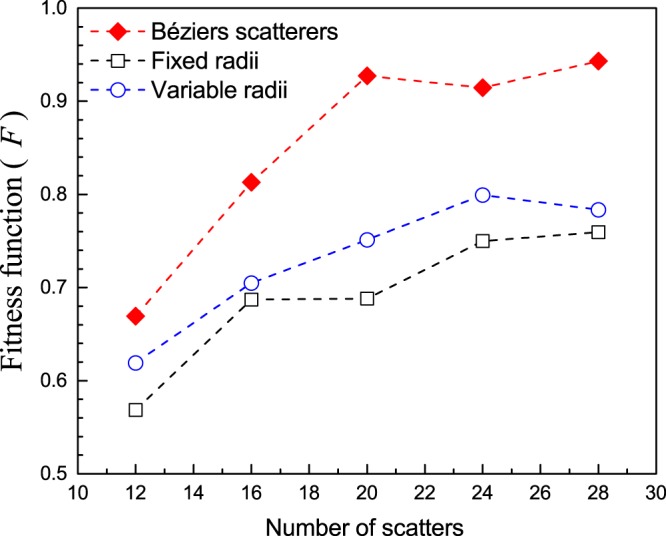
Dependence of the fitness function with the number of scatterers contained in the cloak. Three type of scatterers have been considered in the optimization procedure: Bézier scatterers (diamond symbols), scatterers with circular sections of equal radii (hollow squares) and scatterers with circular sections but variable radii (hollow circles). *F* = 1 means perfect concealing of the central object.

Figure 2 reports the values of the fitness function, *F*, obtained for cloaks containing 12, 16, 20, 24 and 28 discrete scatterers. It is observed that, for the same number of scatterers, the cloaks based on Bézier scatterers are more efficient in accomplishing the concealing condition embedded in Eq. 3. It is concluded that the cloak with 20 Bzier scatterers represents the solution with the minim set of discrete elements and simultaneously providing the maximum value of F. However, it is possible that the number of discrete element could be further reduced by using additional DOF in the definition of the individual elements; for example, by removing the condition of equal shape. The parameters of the five scatterers determining the optimum cloak are given in Table 2. In addition, *r*_0_ = 0.375 cm has been chosen as the fixed value employed in the construction of the six control points defining the profile of a given Bézier scatterers. The positions and shape of the rest of scatterers, up to 20, are obtained by applying the two mirror symmetry operations; one with respect to *x* = 0 and the other with respect to *y* = 0. Regarding computational effort, the CPU time required to obtain the optimum Beziers cloak based on 20 scatters has been reduced in about 90% in comparison with the time required to obtain the optimum cloak made of 120 circular-shape scatters.

**Table 2:** Parameters of the five Bézier scatterer defining the optimum cloak. A given scatterer is centered at the position with coordinates (*x*, *y*). Note that all the scatterers have the same shape parameters, *a*_*i*_. The rest of scatterers forming the cloak are obtained by symmetry operations.

Scatterer Bézier	*x* (cm)	*y* (cm)	*a*_1_	*a*_2_	*a*_3_	*β*	*θ* degrees
1	3.40	5.59	0.278	0.295	0.360	2.401	319.0°
2	7.00	6.34	0.278	0.295	0.360	3.980	162.8°
3	10.59	2.77	0.278	0.295	0.360	2.313	0.2°
4	3.23	9.51	0.278	0.295	0.360	3.295	177.1°
5	7.75	1.98	0.278	0.295	0.360	4.945	237.5°

**Figure 3:**
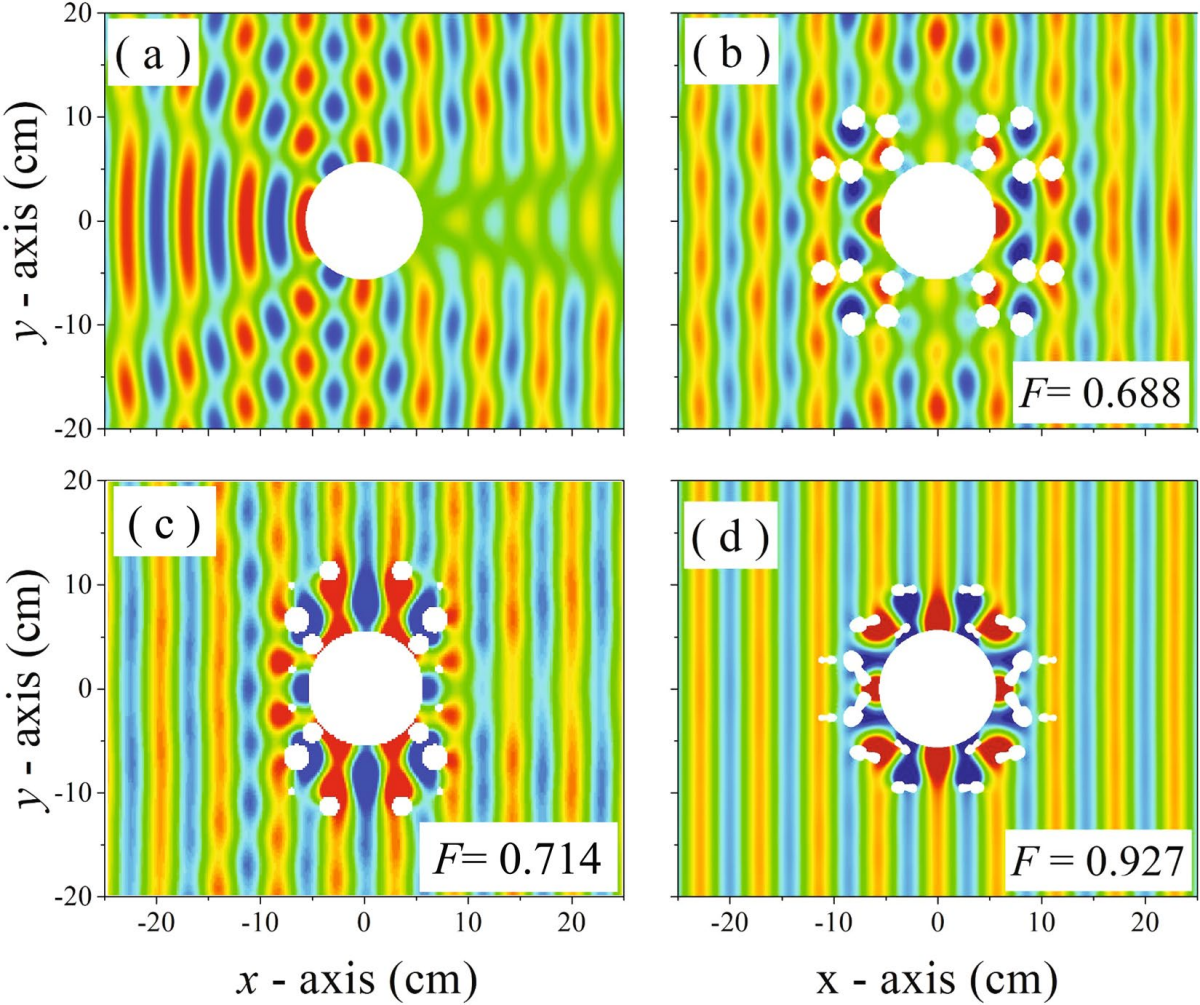
Total pressure maps obtained at 6 kHz from the interaction of a plane wave with: a cylindrical rigid object (**a**); the object with a cloak made of 20 circular cylinders with equal radii (**b**); the object with a cloak made with 20 cylinders with different radii (**c**); the object with a cloak made of 20 Bézier scatterers (**d**).

Figure 3(a) shows the total pressure map resulting from the interaction of an incident plane wave with the bare obstacle, which strongly distorts the pressure field. In comparison, Fig. 3(b),(c) and (d) plots the total pressure maps obtained when the obstacle is surrounded by the three different cloaks resulting from the optimization process. It is clearly shown that for the cloaks based on circular scatterers, both with *F* = 0.679, the reconstruction of the incident plane wavefront is imperfect, either in reflection as well as in transmission. However, for the cloak based on Bézier scatterers [see Fig. 3(d)] the reconstruction of the plane wavefront is almost perfect since *F* = 0.927.

The origin of the large difference obtained for the scattering cancellation condition is attributable to the large increase of DOF when Bézier scatterers are considered in the optimization process. Cloaks based on circular scatterers with similar cancellation efficiency could be obtained but paying the cost of adding a large number of scatterers in the cloak. For example, a cloak made of 120 circular scatterers was designed and experimentally proven in ref.^14^. On the other hand, without experimental support, a cloak made of 78 circular scatterers containing three different radii was proposed in ref.^16^. In physical terms, the result obtained can be understood by considering that the scattered field by a simple circular-shape-scatterer shape is dominated by monopole scattering while a scatterer with an arbitrary external profile introduces higher scattering orders, making possible more efficient cloaks with less number of scatterers. It must be also point out that viscothermal effects are not an issue for these cloaks based on scatterers with rounded shapes, as it is the case of scatterers defined by circular and Béziers curves. Numerical simulations recently performed in the framework of the Boundary Element Method and Finite Element Method predict that viscothermal effects slightly decrease the scattered signal; in an amount of about 5% to 7%^28^.

**Figure 4:**
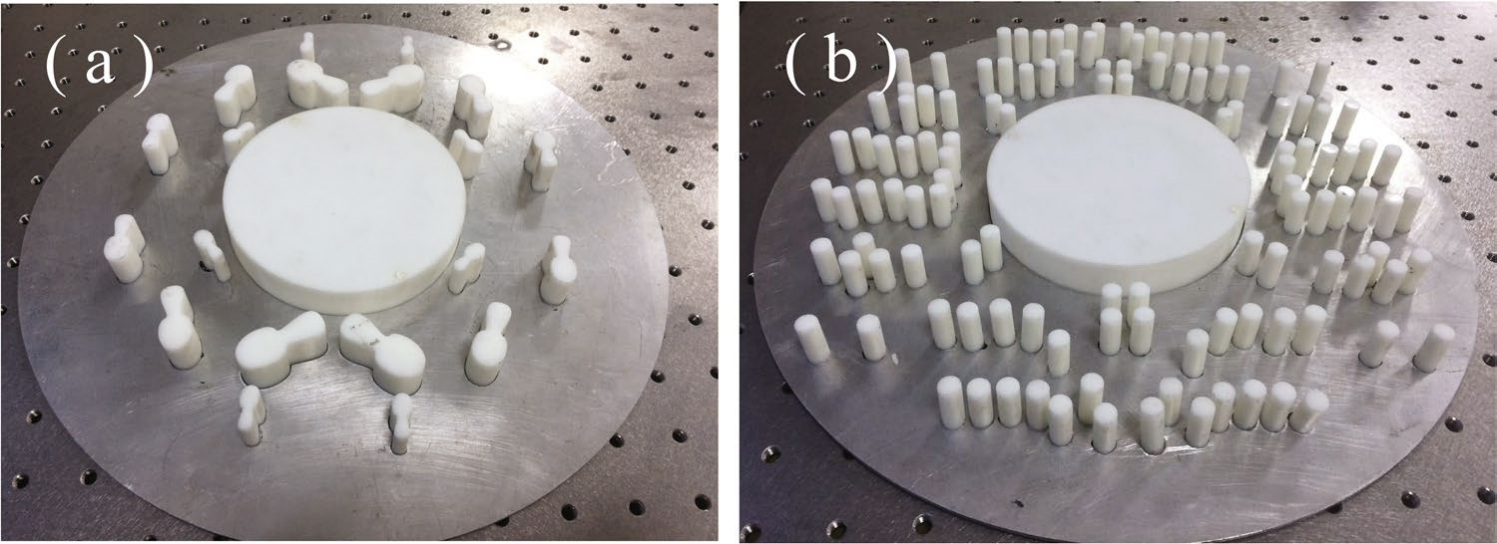
Photographs of the structures fabricated with a 3D printer in ABS plastic. (**a**) Object plus cloak made of 20 Bézier cylindrical scatterers; (**b**) object plus cloak made of 120 cylindrical scatterers with circular cross-section.

Two cloak samples were fabricated in a 3D printer that uses ABS plastic as building material. Photographs of both cloaks are shown in Fig. 4. Figure 4(a) represents the cloak made of 20 Bézier scatterers designed above. The cloak shown in Fig. 4(b), consisting of 120 circular scatterers, were designed in ref.^14^ and has been also characterized for the sake of comparison using the same experimental setup.

We conducted a series of measurements around the selected working frequency in order to characterize the performance of the cloak. As the parameter quantifying the cloaking performance we use the averaged visibility of the object (*γ*), which is defined as^6,14^.

4 $$\gamma (\omega )=\frac{1}{N}\sum _{j}\frac{|{P}_{max,j}|-|{P}_{min,j}|}{|{P}_{max,j}|+|{P}_{max,j}|},$$

where *P*_*max*_ and *P*_*min*_ are the maximum and minimum peak values within a given wavefront *j* and *N* is the total number of wavefronts inside the scanned area.

**Figure 5:**
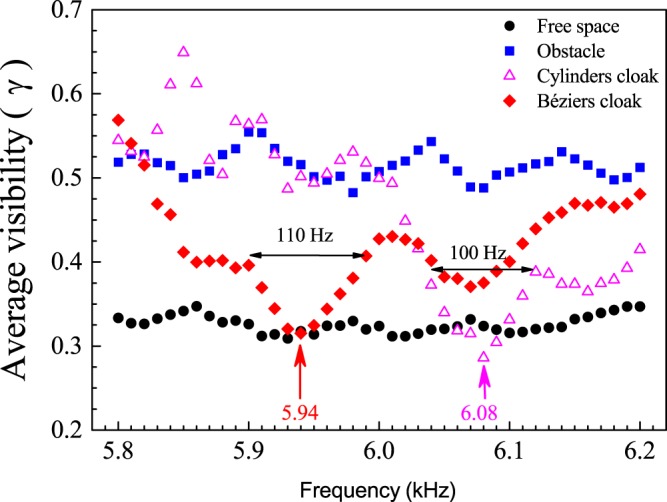
Frequency dependence of the averaged visibility (*γ*) associated to sound waves propagating in free space (black dots) and the waves interacting with a rigid circular obstacle placed at the center of the waveguide (square symbols). The diamond symbols (hollow triangles) represent the data measured when the obstacle is surrounded by the cloak made of 20 Bézier scatterers (120 circular cylinders). The acoustic cancellation occurs at 5.94 kHz and 6.08 kHz for the case of the Bézier cloak and cylinder cloak, respectively.

Figure 5 depicts the frequency dependence of *γ* for the two manufactured cloaks: the one made of circular scatterers (hollow triangles) and that made of Bézier scatterers (diamond symbols). The figure also includes the characterization, in terms of *γ*, of the propagation in the empty WG (free space) and for the WG containing the bare obstacle. The cloaks reach minimum values of *γ* at the frequencies of 5940 Hz and 6080 Hz, respectively. The discrepancy of these frequencies with 6000 Hz, the target frequency employed in the design procedure, is due to the different sound speeds existing inside the WG at the time when the characterization was performed. A small deviation in the ambient temperature and humidity conditions explain the small, of about ±1%, discrepancy in the frequency at which the averaged visibility takes its minimum value. The average visibility of both cloak at their minimum value coincides with that obtained for the empty WG, supporting the claim that the condition od acoustic cancellation has been achieved. To be more precise, Fig. 5 shows that the minimum of *γ* for the case of the cloak made of circular cylinders is below the value measured for the empty waveguide. This result should be interpreted in terms of the experimental error associated with the experimental setup rather to absorption losses. Particularly, for the Bézier cloak, the operational bandwidth is about 110 Hz while for the circular-shape cloak is around 100 Hz.

**Figure 6:**
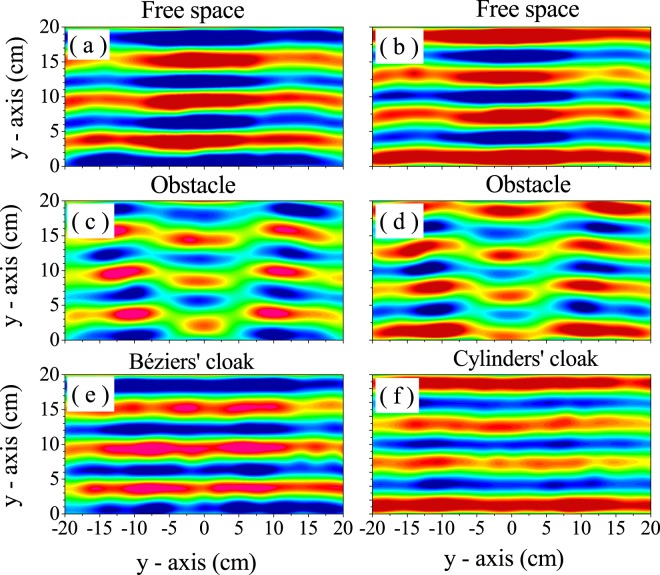
Total pressure maps (real part) measured at 5940 Hz (left panels) and 6080 Hz (right panels). (**a**) and (**b**) represent the maps obtained when the waveguide is empty (free space). (**c**) and (**d**) show the maps obtained with the obstacle inserted in the waveguide. Finally, (**e**) and (**f**) plot the maps resulting from the designed cloaks surrounding the obstacle. Both cloaks practically recover the plane wavefronts depicted in (**a**) and (**b**), respectively.

A further support of this claim is shown in Fig. 6, which shows the total pressure maps measured for the different situations under study. Recorded data at 5940 kHz (left panels) and 6080 Hz (right panels) are depicted within the scanned region in the experimental setup. At these frequencies, the averaged visibility of the fabricated cloaks have the minimum values of *γ*, which are almost equal to that obtained for the empty WG. Therefore, it is concluded that a perfect concealing of the obstacle has been achieved within our experimental setup. The pressure map shown in Fig. 6(e) validates the designed cloak based on Bézier scatterers since it reconstructs the pressure map in 6(a), corresponding to free propagation inside the waveguide. The quality of this reconstruction is clearly noticed if we observe the pressure map shown in Fig. 6(c), corresponding to the case of the bare obstacle. In addition, we can also conclude that a cloak based in solely 20 Bézier scatterers has a performance equal to the cloak made of 120 circular scatterers^14^. These conclusions are encouraging since they open the possibility of tackling the design of omnidirectional 2D cloaks based on a reasonable number of discrete scatterers. The design of 3D cloaks based on 3D Bézier scatterers is also a natural extension of the approach here introduced.

## Discussion

In summary, we have shown that cloaks based on scattering cancellation are feasible by using a reduced number of discrete scatterers. These cloaks are achieved by adding degrees of freedom in the definition of the individual scatterers forming the cloak. With this purpose, we have introduced a new type of scatterers, named *Bézier scatterers*, whose external shape is obtained by a combination of two cubic Bézier curves. The positions and shape of the Bézier scatterers result from an optimization procedure imposing the condition of scattering cancellation. A cloak made of solely 20 Bézier scatterers has been designed and experimentally demonstrated. We have demonstrated experimentally that this minimum cloak operates within a narrow frequency band centered at 5.94 kHz, with a bandwidth of about 110 Hz. It has been also shown both theoretically and experimentally that its performance, in terms of averaged visibility, is similar to cloaks made of 120 cylindrical scatterers previously reported in ref.^14^. We should point out that the number of discrete elements in the cloak can be further reduced by removing the condition of equal shape for all the Bézier scatterers or even performing a complete optimization of the scatterers shape; i.e., not restricted to the shape provided by the Bézier curves. The possibility of having strong losses due to viscothermal effects should be tackled at the same time, by developing an optimization algorithm in which the shapes of the elements of the cloak would create minimum losses.

The results here obtained are encouraging since they open the possibility of overcome the two drawbacks associated to these types of cloaks, that is directionality and narrow band operation. Both properties can be resolved by adding degrees of freedom to the design procedure, which should be based on a multi-objective optimization. The extension of the method here introduced to design 3D cloaks is also possible by developing scatterers Bézier in three dimensions.

## Methods

### Solution of the scattering problem

The temporal dependence *e*^−*iωt*^ is implicit in all the derivations that follows but will be omitted for simplification. The Helmholtz equation in 2D defines the scattering problem to be solved,

5 $${\nabla }^{2}{p}^{sc}(r,\theta )+{k}^{2}{p}^{sc}(r,\theta )=0,$$

where *p*^*sc*^(*r*, *θ*) is the scattered pressure at any arbitrary point (*r*, *θ*) in the space outside of the scatterers. *k* represents the wavenumber defined by *k* = *ω*/*c*, where *ω* is the angular frequency (*ω* = 2*πν*) and *c* is the sound speed in air. For the calculations, we consider that *c* = 343 m/s.

The equation above has been solved with the method of fundamental solutions (MFS)^32^. The MFS is a meshfree boundary collocation method which is applicable to boundary value problems in which a fundamental solution of the operator in the governing equation is known explicitly^33^. In MFS, the solutions of the problem in question are approximated by a linear combination of fundamental solutions of the Helmholtz equation. The method is especially suitable for axisymmetric configuration of scatterers^34^, as it is here.

Let us consider an incident plane wave propagating along the *x*-axis, *p*^*in*^ = *e*^*ikx*^. This impinging wave produces a total scattered pressure given by,

6 $${p}^{sc}(r,\theta )=\sum _{j=1}^{N}{\alpha }_{j}G(r,\theta ;{r}_{j}^{\prime} ,{\theta }_{j}^{^{\prime} })$$

where *α*_*j*_ are the complex coefficients to be determine and $G(r,\theta ,{r}_{j}^{\prime} ,{\theta }_{j}^{^{\prime} })=\frac{i}{4}{H}_{0}^{(1)}(kR)$ is the fundamental solution of the 2D Helmholtz equation. ${H}_{0}^{\mathrm{(1)}}$ denotes the Hankel function of first kind and order zero, *R* is the distance between the source and sample points and the imaginary number $i=\sqrt{-1}$. By choosing *N* sample points (*r*_*i*_, *θ*_*i*_) on the surface of scatterers and imposing the Neumann’s boundary conditions to the total field, ${p}^{to}={p}^{in}+{p}^{sc}$, we arrive at a linear system of *N* equations for the unknowns coefficients *α*_*j*_,

7 $${[\frac{\partial {p}^{in}}{\partial n}]}_{({r}_{i},{\theta }_{i})}+\sum _{j=1}^{N}{\alpha }_{j}{[\frac{G(\overrightarrow{r},{\overrightarrow{r}}_{j}^{^{\prime} })}{\partial n}]}_{({r}_{i},{\theta }_{i})}=0,\,i=1,\ldots ,N$$

where $\overrightarrow{n}$ is the normal to the surface at the selected point. However, the unknown coefficients are not accurate for scatterers with arbitrary shape. Our analysis lead us to conclude that the key point is the definition of the positions ${\overrightarrow{r}}_{i}$ at which the derivative ∂*p*^*in*^/∂*n* has to be calculated. Often, the ${\overrightarrow{r}}_{i}$ is regarded as the radius vector between the sample points and the geometric center. But actually, ${\overrightarrow{r}}_{i}$ should be taken as a vector contained in a direction normal to the surface. We can get the correct result for an arbitrary shape scatterer by using the correct definition of *r*_*i*_. Once the scattering problem is solved, the fitness function defined in the optimization method can be calculated for each iteration step.

### Experimental setup and procedure

**Figure 7:**
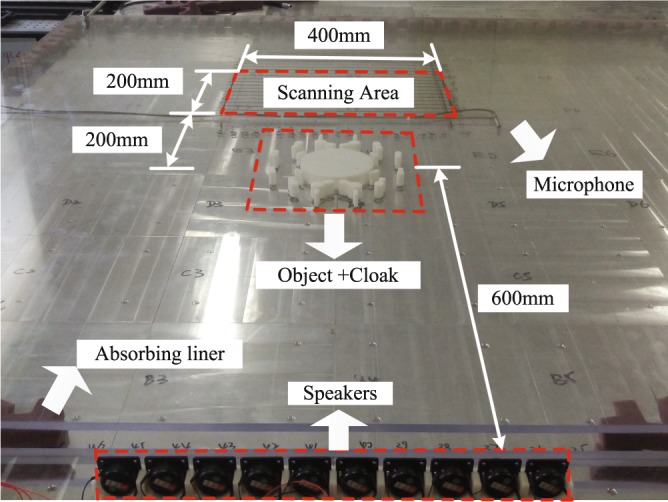
Photograph of the experimental setup with some dimension of interest.

The cylindrical bare object and the object with the designed cloaks have been characterized in a 2D acoustic waveguide (WG) with dimensions 1.5 × 1.5 × 0.02 m^3^. A photograph of the WG and part of the experimental setup is shown in Fig. 7. The upper and bottom surfaces of the waveguide are made of two Perspex sheets with equal thickness. The frontal face of the waveguide has an aperture with dimension 0.4 × 0.05 m^2^ where an array of loudspeakers produce the incident sound that propagates inside the waveguide. In order to avoid undesired reflections, a 5 cm layer of fiberglass covers the lateral and rear faces of the WG. Due to small height of the waveguide, no modes with oscillations along *z*-axis are expected below 8500 Hz, the cut-off frequency at which half-wavelength equals the chamber height. Therefore, the 2D approach employed in the design process can be considered valid within this frequency range. A programmable robot put at the rear face of the WG moves a G.R.A.S 46BE microphone, with scans the area of 2 × 40 cm^2^ located behind the object and the cloak. A spatial resolution of 2 cm in employed in each direction. The excitation signal is represented by 6000 Hz sine pulses. Six repeated pulses are emitted at each position of the robot, and the resulting data are processed and stored in a computer.

## Data Availability

The authors declare that all data supporting the results of the study are available within the published results.
